# Preventing Bakri Balloon Puncture During Cesarean Section in Placenta Previa: Fundal Fixation Temur Technique

**DOI:** 10.7759/cureus.96038

**Published:** 2025-11-03

**Authors:** Muzaffer Temur, Süleyman Serkan Karasin

**Affiliations:** 1 Obstetrics and Gynecology, Faculty of Health Sciences, İstanbul Kent Üniversitesi, Beyoğlu, TUR; 2 Obstetrics and Gynecology, Bursa Yüksek İhtisas Training and Research Hospital, Bursa, TUR

**Keywords:** bakri balloon, childbirth, postpartum hemorrhage, surgical technique, uterine atony

## Abstract

Placenta previa is a major risk factor for obstetric hemorrhage. Bakri balloon tamponade (BBT) is a well-established uterine-sparing method, but balloon puncture during uterine closure may compromise its effectiveness. We present a fundal fixation technique, a simple modification involving temporary fundal fixation of the balloon catheter to displace it away from the hysterotomy site during closure. This technical report outlines the method and summarizes the outcomes from four cases of placenta previa that were managed successfully. This fundal fixation technique is a safe and effective method for minimizing balloon puncture risk during cesarean sections.

## Introduction

Placenta previa is a major cause of obstetric hemorrhage and maternal morbidity. It occurs when the placenta partially or completely covers the internal cervical os, obstructing the birth canal and predisposing to profuse bleeding during uterine contractions or surgical incision. The bleeding mechanism is mainly related to the disruption of the low-lying placental attachment as the lower uterine segment stretches in late pregnancy or during delivery. The reported incidence ranges from 3 to 5 per 1000 pregnancies, with increasing frequency due to higher cesarean delivery rates and assisted reproductive technologies [1,2]. Severe hemorrhage in placenta previa often necessitates surgical interventions such as uterine artery ligation, pelvic embolization, or even hysterectomy [3]. To avoid these radical procedures and preserve fertility, intrauterine balloon tamponade devices, particularly the Bakri balloon tamponade (BBT), have become a first-line conservative management tool. Introduced in 2001 by Bakri et al., the balloon exerts uniform pressure within the uterine cavity to compress bleeding vessels and promote hemostasis [4]. The standard technique involves inserting the balloon through the uterine incision or cervix and inflating it with sterile fluid until bleeding subsides.

Despite its effectiveness, several practical challenges remain. One of the most significant is balloon puncture or rupture during closure of the uterine incision in cesarean deliveries complicated by placenta previa. Such an event compromises tamponade effectiveness and may lead to recurrent bleeding and additional blood loss [5]. Previous researchers have proposed various modifications to improve balloon stability and safety. For example, Kumru et al. suggested inserting the balloon only after uterine closure to prevent puncture; however, this approach delays hemostasis and increases the risk of bacterial contamination [6]. Matsubara et al. developed cervical-holding and abdominal traction stitch techniques to prevent balloon slippage; however, these do not eliminate the risk of puncture [7,8]. Consequently, a reliable and simple preventive measure remains lacking.

The Fundal Fixation Temur Technique was developed to address this issue. By temporarily anchoring the Bakri balloon catheter to the uterine fundus, the device is displaced away from the hysterotomy site during closure, minimizing the risk of injury and maintaining effective tamponade. This report presents the technical steps of the fundal fixation method and summarizes its successful application in four cases of placenta previa managed during cesarean section.

## Technical report

**Figure 1:**
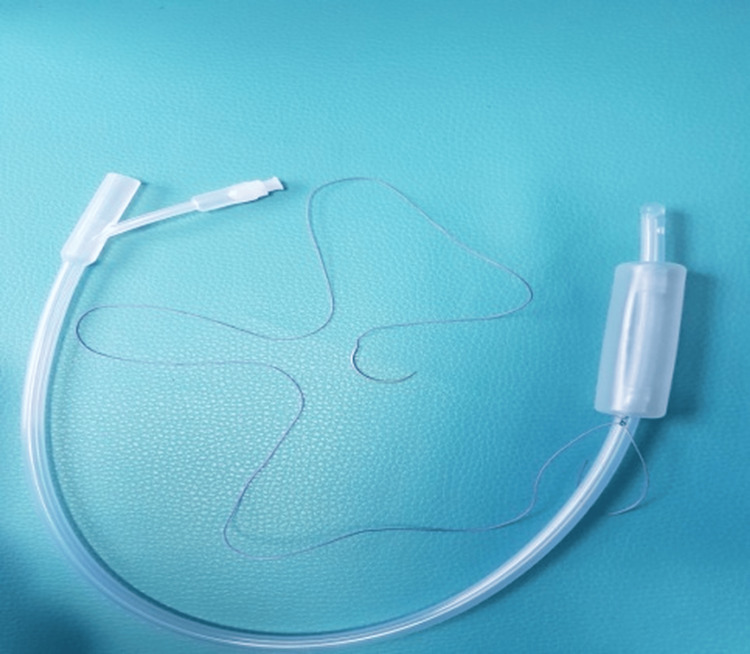
Photograph of the Bakri balloon showing silicone body (24 Fr), drainage port, and inflation channel. The balloon is designed for intrauterine tamponade with a maximum fill volume of 500 mL. In this report, 160-220 mL sterile water was used to achieve adequate lower-segment pressure. Bakri balloon (Bloomington, IN: Cook Women’s Health)

**Figure 2:**
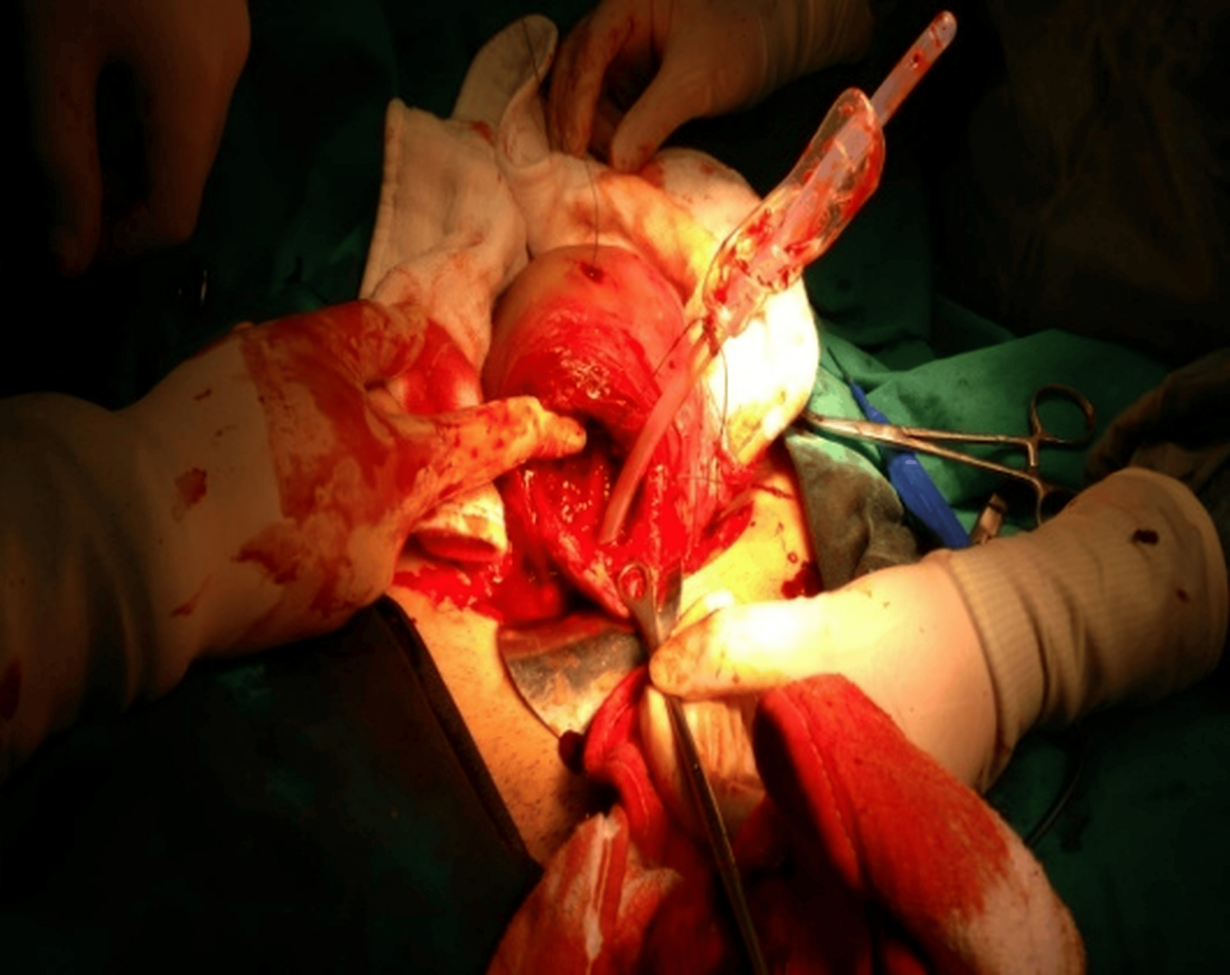
After placenta removal, a #1 absorbable polyglactin suture was tied around the mid-portion of the Bakri catheter. The needle was passed from the uterine cavity to the serosal surface of the fundus approximately 2 cm above the upper margin of the hysterotomy incision, at a 30-45° upward angle, ensuring the balloon remained below the suture path.

**Figure 3:**
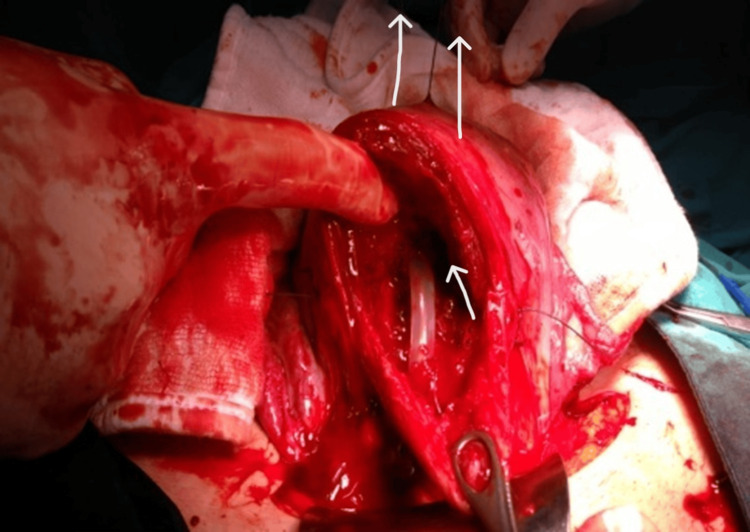
Upward retraction of the balloon. Gentle manual traction (approximately 0.5 kg force) was applied to the fundal suture, elevating the balloon toward the fundus and displacing it away from the incision site. The uterus was closed under direct vision while maintaining traction. Arrows indicate the direction of suture tension.

**Figure 4:**
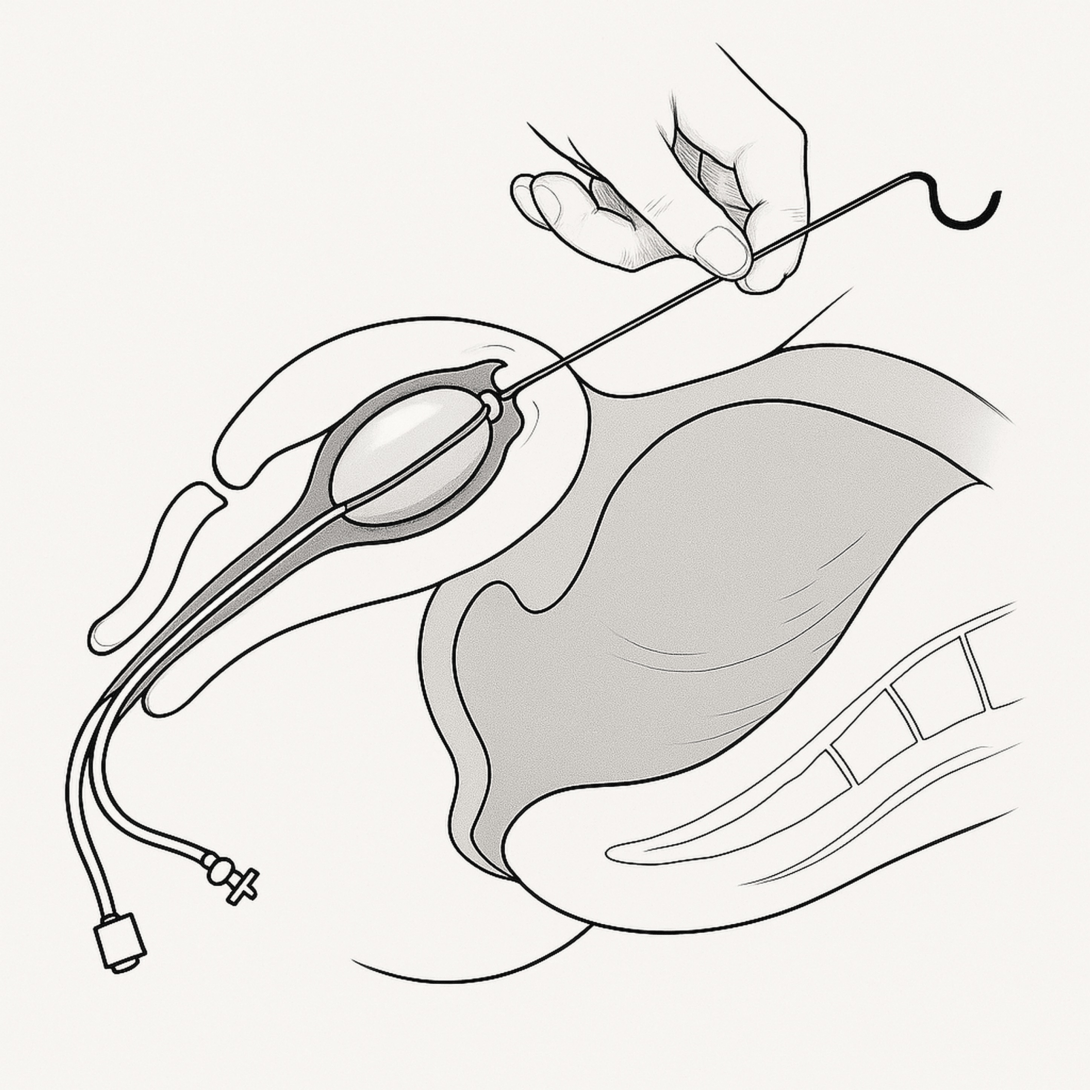
Schematic illustration of Fundal Fixation Temur Technique. Graphic showing uterine anatomy and final balloon position. The Bakri balloon occupies the lower uterine segment and cervix, while the fixation suture exits through the fundal serosa. The distance between the balloon and hysterotomy incision is approximately 2-3 cm, preventing needle puncture during closure. This image is created by the authors of this study. Bakri balloon (Bloomington, IN: Cook Women’s Health)

Four women with placenta previa underwent cesarean section at Bursa Yüksek İhtisas Training and Research Hospital between January 2023 and May 2024. Placenta previa was diagnosed preoperatively by ultrasonography and classified as major or minor. After delivery and removal of the placenta, a Bakri balloon (Bloomington, IN: Cook Women’s Health) was introduced through a uterine incision (Figure 1). A #1 polyglactin suture was tied around the mid-portion of the catheter. The attached needle was passed from the uterine cavity through the fundus, allowing the balloon to be retracted upward into the uterine fundus under gentle traction (Figure 2). This maneuver displaced the balloon away from the incision line, allowing safe closure without puncture (Figures 3, 4). The balloon was inflated with 160-220 mL of sterile water under direct vision while maintaining traction. After closure, the fundal suture was cut close to the surface. Placement was confirmed by ultrasound, and blood loss was monitored through the drainage port. No vaginal gauze packing was required. The outcomes are summarized in Table 1.

**Table 1:** Demographic and clinical data of four cases. Prev: previous; C/S: cesarean section; y: year; GA: gestational age; wks: weeks; U: unit; ES: erythrocyte suspension; FFP: fresh frozen platelets

Case	Age (years)	Prev. C/S	GA (wks)	Previa type	Blood loss (mL)	Transfusion	Hospital stay (days)
1	26	1	37+0	Major (anterior)	1320	2U ES	5
2	38	1	32+5	Major (posterior)	1400	2U ES + 2U FFP	4
3	24	1	37+4	Major (posterior)	950	None	3
4	32	2	38+0	Minor (anterior)	1000	None	3

## Discussion

Bakri balloon tamponade (BBT) has been shown to be highly effective in controlling postpartum hemorrhage, with reported success rates ranging from 75% to 90% [9,10]. Its hemostatic effect depends on uniform intrauterine pressure, which compresses the bleeding vessels and promotes myometrial contraction. However, in cesarean deliveries complicated by placenta previa, the proximity of the lower uterine segment incision to the balloon body introduces a practical limitation, i.e., balloon puncture or rupture during hysterotomy closure. Once punctured, the balloon immediately loses tamponade function, leading to uncontrolled bleeding and the potential need for invasive interventions [5].

Several strategies were developed to address this issue. Kumru et al. proposed inserting the balloon only after closure of the uterine incision, which indeed eliminates puncture risk but delays hemostasis and increases the risk of contamination due to transvaginal manipulation [6]. Matsubara et al. suggested cervical-holding and abdominal traction stitch methods to prevent balloon prolapse into the vagina; however, these techniques do not solve the problem of puncture during closure [7,8]. Maher and Abdelaziz compared balloon and non-balloon management protocols, emphasizing safety during insertion but not addressing the risks of mechanical injury [5].

Recent studies continue to explore ways to stabilize balloon placement and prevent displacement or leakage. Cassardo et al. reviewed preventive strategies for balloon displacement and highlighted the need for simple, reproducible mechanical solutions [11]. Similarly, Liu et al. demonstrated that combining balloon tamponade with supportive suturing techniques enhances hemostasis in complicated cesarean deliveries; however, these techniques require additional procedural steps [12].

The Fundal Fixation Temur Technique provides a straightforward modification that directly eliminates puncture risk without delaying tamponade. By passing a single absorbable suture through the uterine fundus and applying gentle traction to elevate the Bakri balloon, the device is displaced away from the incision site. This maneuver maintains uninterrupted pressure in the lower uterine segment while creating a safe surgical field for closure. In addition, upward traction reduces the risk of balloon prolapse, eliminating the need for vaginal packing and facilitating postoperative monitoring.

In the four cases discussed here, fundal fixation ensured stable balloon positioning, effective hemostasis, and acceptable blood loss, without the need for additional interventions. The method is simple, cost-free, and easily reproducible. Its main limitation is the small sample size; however, our experience supports its safety and practicality. Larger, multicenter studies are warranted to validate the reproducibility and long-term outcomes of this approach. Future comparative studies are planned to evaluate this technique against the standard Bakri balloon application in a larger cohort.

In conclusion, the Fundal Fixation Temur Technique represents a novel, practical, and safe modification that complements previously reported strategies by specifically preventing balloon puncture during uterine closure [6-12]. This simple maneuver may enhance the reliability of Bakri balloon use in cases of placenta previa and deserves further evaluation in larger series.

## Conclusions

The Fundal Fixation Temur Technique represents a simple intraoperative modification designed to reduce the risk of Bakri balloon puncture during cesarean section for placenta previa. Based on four illustrative cases, the technique appeared technically feasible, safe, and easily applicable without additional equipment. In all cases, hemostasis was achieved with the Bakri balloon alone, and no balloon-related complications occurred.

However, given the small number of cases and the absence of a control group, no conclusions can be drawn regarding comparative efficacy, blood loss reduction, or generalizability to other causes of obstetric hemorrhage. The present report should therefore be interpreted as an initial technical description rather than as evidence of effectiveness. Future prospective and comparative studies involving larger cohorts and multiple surgeons are needed to validate the reproducibility, safety, and potential benefits of this modification.
